# *Salmonella typhimurium* Infection Reduces *Schistosoma japonicum* Worm Burden in Mice

**DOI:** 10.1038/s41598-017-00992-1

**Published:** 2017-05-02

**Authors:** Xiaoyang Zhu, Lu Chen, Junfang Wu, Huiru Tang, Yulan Wang

**Affiliations:** 1 CAS Key Laboratory of Magnetic Resonance in Biological Systems, State Key Laboratory of Magnetic Resonance and Atomic and Molecular Physics, National Centre for Magnetic Resonance in Wuhan, Wuhan Institute of Physics and Mathematics, the Chinese Academy of Sciences, Wuhan, 430071 China; 2 State Key Laboratory of Genetic Engineering, Zhongshan Hospital and School of Life Sciences, Fudan University, Collaborative Innovation Centre for Genetics and Development, Shanghai International Center for Molecular Phenomics, Shanghai, 200433 China; 3 Collaborative Innovation Center for Diagnosis and Treatment of Infectious Diseases, Zhejiang University, Hangzhou, 310058 China

## Abstract

Coinfection of microorganisms is a common phenomenon in humans and animals. In order to further our understanding of the progress of coinfection and the possible interaction between different pathogens, we have built a coinfection mouse model with *Schistosoma japonicum* and *Salmonella typhimurium*, and used this model to investigate the systemic metabolic and immune responses using NMR-based metabonomics and immunological techniques. Our results show that *Salmonella typhimurium* (ATCC14028) infection reduces the number of adult schistosomal worms and eggs, relieves symptoms of schistosomiasis and also abates the mortality of mice infected by *Schistosoma japonicum*. In addition, *Salmonella typhimurium* infection counteracts the metabolic disturbances associated with schistosomiasis, which was reflected by the reverted levels of metabolites in coinfected mice, compared with the *Schistosoma japonicum* infected mice. Furthermore, immune analyses also indicate that shift of the immune response to different pathogens is a result of indirect interactions between *Schistosoma japonicum* and *Salmonella typhimurium* within the host. *Salmonella typhimurium* infection can ameliorate *Schistosoma japonicum*-caused schistosomiasis in BALB/c mice, which is most likely due to inverse immune polarization. Our work provides an insight into coinfection between *Schistosoma japonicum* and *Salmonella typhimurium*, and may further contribute to the development of new tools for controlling *Schistosoma japonicum-*associated diseases.

## Introduction

Coinfection with diverse pathogens (e.g. virus, bacterium, fungus, protozoan and helminth) is a common occurrence in humans^1–4^, and this is estimated to exceed one sixth of the global population^5^. People infected with multiple helminths reached over 800 million^6^, mostly of which occurred in developing countries^7^. Other microorganisms, including *Salmonellae* ^8^, *Mycobacterium tuberculosis* ^9^, *Helicobacter pylori* ^10^, human immunodeficiency virus^11^, hepatitis C virus^12^, dengue virus^13^ etc, are also involved in coinfections. Compared to infection caused by one single pathogenic species, coinfection can often alter the pathogenicity of infectious diseases^14, 15^, availability of hosts^16^, clinical severity^17^, and thus influence the prevention and control of pathogen-associated diseases^18^. Therefore, further understanding on the mechanisms of coinfection is greatly needed.

Schistosomiasis is one of the most debilitating and widespread human diseases caused by infection with a parasitic blood fluke called Schistosome. Schistosome infection causes severe damage to the host and can eventually lead to death^19^. Coinfection of Schistosome and other pathogens is also frequent in epidemic areas^20, 21^ where people are exposed to helminths and other bacterial pathogens, such as *Mycobacterium tuberculosis* ^22^, or *Salmonella* ^8^. Although numerous studies have sought to uncover the interactions and mutual influences between these concurrent pathogens^12, 23, 24^, little is known regarding how the host response to these concurrent infections. Previously, we investigated the metabolic effects of hamsters coinfected with *Schistosoma japonicum* (*S. japonicum*) and *Necator americanus* ^25^. We found that coinfection, surprisingly, had no impact on the worm burden and that metabolic response of the host was sum of the two-single infections^25^. This could be due to both parasites being introduced to the host simultaneously, thereby removing any competition for energy resources or growth. It may also be possible that distinct immune responses stimulated by either helminth failed to suppress the other pathogen’s growth. Therefore in this study, we aimed to assess the impact of sequential infection of helminths and bacteria on the host. We chose the coinfection of *Salmonella typhimurium* (*S. typhimurium*) and *S. japonicum* as both pathogens are widespread in developing countries^26, 27^, therefore reflecting the high likelihood of concurrent *Salmonellae* and Schistosome infections^28–31^. Schistosome infection can result in a shift of host immune response from Th1 to Th2 polarization, which is in parallel with the progress of the schistosomiasis, including cercariae intruding, larvae migrating, the adult pairing and laying eggs^19^, whereas *S. typhimurium* infection only induces Th1 polarization^32^. Therefore immune interaction between these two species in the same host could be an important clinical consideration.

Evidence from previous investigations have shown that metabonomics is a robust tool for studying the metabolic responses to stimuli^33^, such as from a coinfection^25^. Metabonomics utilizes ^1^H nuclear magnetic resonance (NMR) spectroscopy or chromatography coupled to mass spectrometry, with multivariate statistical analysis for detecting metabolic changes of a system subjected to stress^34^. In this study, we have applied NMR-based metabonomics and immune techniques to investigate the impact of coinfection with *S. typhimurium* and *S. japonicum* on mice. We infected mice with *S. japonicum*, followed by *S. typhimurium* once schistosomiasis was fully established. We found that coinfection with *S. typhimurium* can ameliorate schistosomiasis in terms of worm burden and metabolic alterations associated with the infection, mostly due to host’s self-immune responses manipulated by the secondary bacterial infection. Our finding mirrored increasingly successful immunotherapy for treating cancers^35^, where the self-immune response of host plays a vital role. Our research provided important information about the impact of two infectious organisms on the host, which could provide an alternative avenue for treating schistosomiasis.

## Results

### Worm burden, egg burden in liver, animal survival rate and histopathology

**Figure 1:**
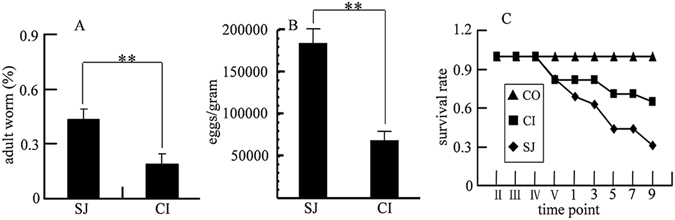
Worm burden, liver egg burden and animal survival rate. (**A**) Adult worm of *S. japonicum* obtained from mice infected with *S. japonicum* (SJ) and those coinfected with *S. japonicum* and *S. typhimurium* (CI); (**B**) *S. japonicum* eggs obtained from liver of mice infected with *S. japonicum* (SJ) and those coinfected with *S. japonicum* and *S. typhimurium* (CI); (**C**) Survival rate of mice in different groups. SJ, mice infected with 80 cercariae of *S. japonicum* only; CI, mice infected with 80 cercariae followed by *S. typhimurium* ATCC14028 infection 5 weeks later; CO, mice as uninfected control. Time points in Roman numerical mean corresponding weekly points, time points in Arabic numerical indicates days after *S. typhimurium* was introduced. The statistically significant differences were calculated using independent samples T test with SPSS 13.0 and error bars represent SEM, **p < 0.01.

**Figure 2:**
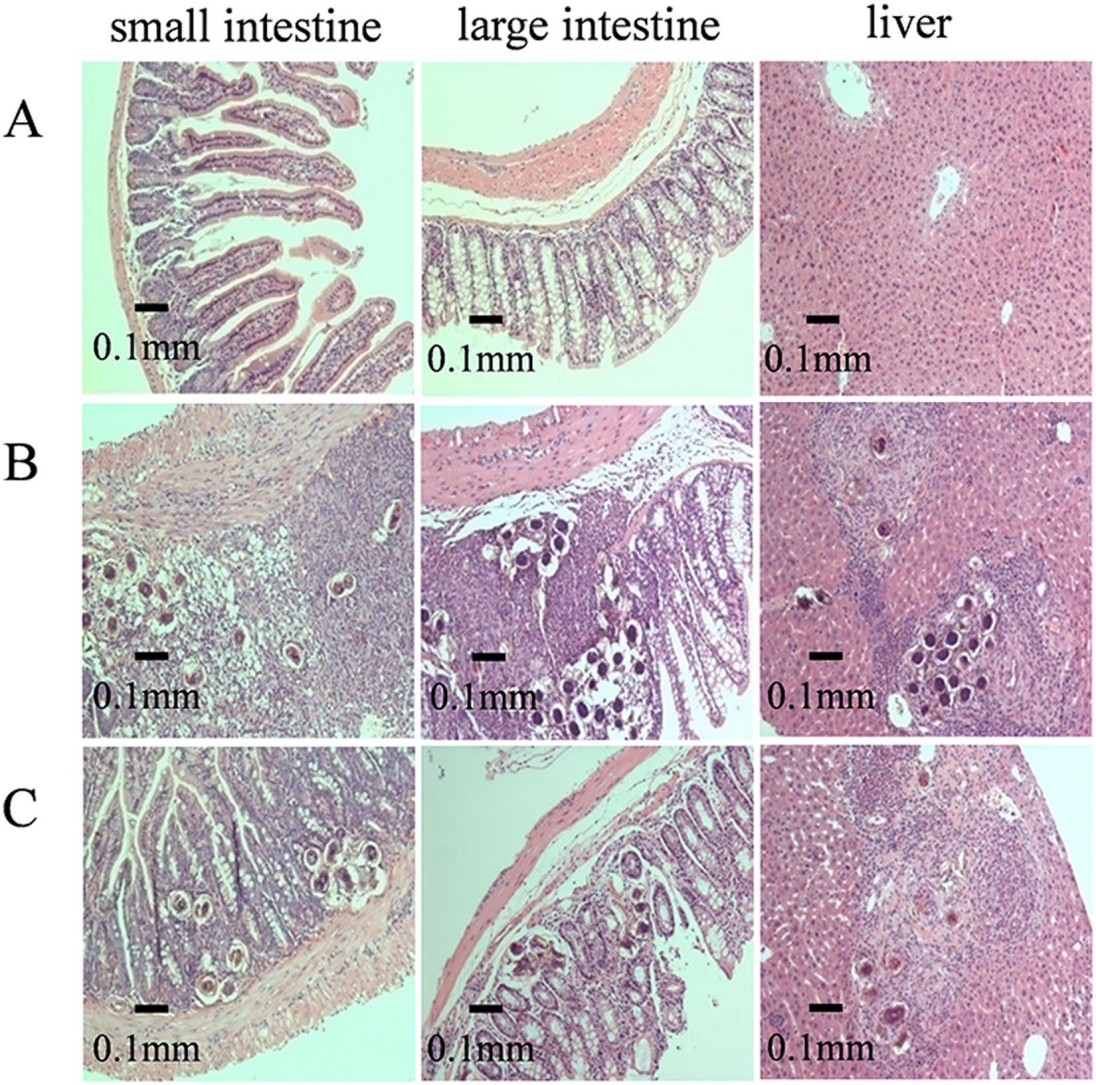
Histopathology examination of intestinal tissues and livers. The panel **A** was from control group, panels **B** and **C** were from single infection of *S. japonicum* and coinfection of *S. japonicum* and *S. typhimurium* respectively.

A total of 19% of adult schistosomes were retrieved from the mice coinfected with *S. japonicum* and *S. typhimurium* (CI), which is significantly lower compared with 44% from mice with *S. japonicum* infection alone (SJ) (Fig. 1A). In addition, a reduction of *S. japonicum* eggs trapped in livers was also found from coinfected mice (Fig. 1B). This observation was confirmed by the histological examination of liver and intestinal tissues (Fig. 2). These data show that mice coinfected with *S. japonicum* and *S. typhimurium* were able to reduce the worm and egg burden of schistosomiasis and as a consequence, increase the survival rate of the coinfected mice. At the end of experiment, i.e. nine days after *S. typhimurium* ATCC14028 inoculation, approximately 70% of mice in the CI group survived and only about 40% survived in SJ group (Fig. 1C).

### Metabolic changes in urine associated with coinfection

In order to identify metabolic changes associated with reduction in worm burden in coinfected mice, we investigated the metabolic profiles of urine, serum and liver extracts of mice from single infected and coinfected groups. A total of nearly 60 metabolites were identified from ^1^H NMR spectra of urine, serum and liver tissue extracts (Supplementary Fig. S1 and Table S1). Metabolic compositions of urine samples were mainly dominated by organic acids, whereas those of serum and liver extracts were dominated by lipids, amino acids, membrane metabolites, glucose and metabolites of RNA and DNA.

**Figure 3:**
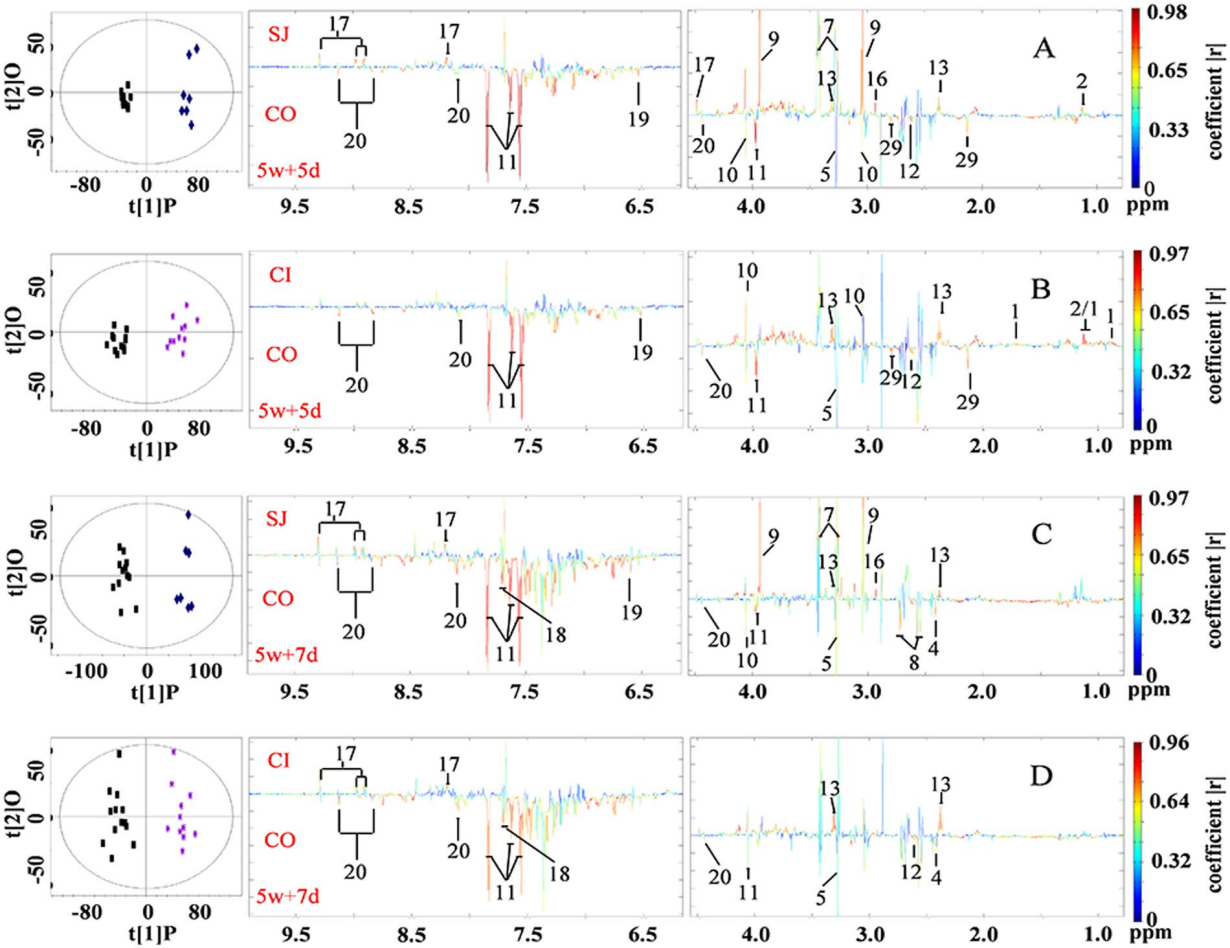
Scores plots and corresponding color-coded coefficient plots. These plots derived from O-PLS-DA of ^1^H NMR spectral data obtained from urine of uninfected control mice (CO), *S. japonicum* infected mice (SJ) and mice coinfected with *S. japonicum* and *S. typhimurium* (CI). Signals upwards indicate increase in the levels of metabolites compared with the control; signals downwards denote a decrease. 5w + 5d, time point, five days after coinfection; 5w + 7d, time point, seven days after coinfection. Keys: 1, 2-keto-3-methy-valerate; 2, 2-keto-isovalerate; 4, succinate; 5, trimethylamine-*N*-oxide (TMAO); 7, taurine; 8, citrate; 9, creatine; 10, creatinine; 11, hippurate; 12, 2-keto-isocaproate; 13, 3-ureidopropionate (3-UP); 16, dimethylglycine (DMG); 17, *N*-methylnicotinamide; 18, indoxyl sulfate; 19, fumarate; 20, trigonelline; 29, *N*-acetylglutamic acid.

We compared urinary metabolic profiles of mice infected with *S. japonicum* and mice coinfected with *S. japonicum* followed by *S*. *typhimurium*, with control urine, at 5 days and 7 days after infection of *S. typhimurium* (i.e. 5 weeks + 5 days and 5 weeks + 7 days). The O-PLS-DA of the metabolic profiles showed that the levels of 13 metabolites had significantly changed after *S. japonicum* single infection (Fig. 3A and Supplementary Table S2) and almost identical changes were noted two days after (Fig. 3C and Supplementary Table S2). These were the elevated levels of 2-keto-isovalerate, *N*-methylnicotinamide (NMN), dimethylglycine (DMG), creatine, creatinine, taurine, 3-ureidopropionate (3-UP) and depleted levels of trigonelline, fumarate, trimethylamine-*N*-oxide (TMAO), hippurate and *N*-acetylglutamic acid. Considerable numbers of metabolites (including taurine, citrate, creatine and DMG) in the coinfected group reverted back to the levels comparable with uninfected control group (Fig. 3B,D and Supplementary Table S2) at both of the time points.

### Metabolic changes in liver associated with coinfection

**Figure 4:**
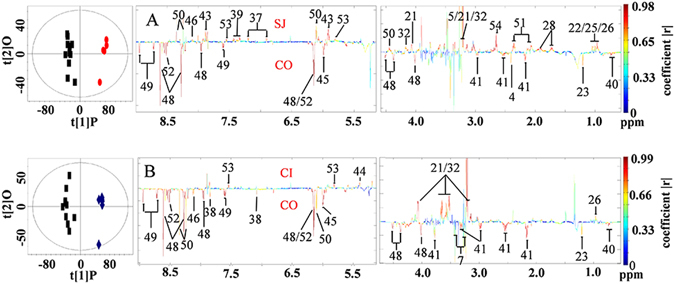
Scores plots and corresponding color-coded coefficient plots. These plots derived from O-PLS-DA of ^1^H NMR spectra of liver extracts obtained from uninfected control mice (CO), *S. japonicum* infected mice (SJ), *S. japonicum and S. typhimurium* ATCC14028coinfected mice (CI). Signals upwards denote increases in the level compared to the control; signals downwards indicate decrease in the levels compared to the control. Keys: 4, succinate; 5, trimethylamine-*N*-oxide (TMAO); 7, taurine; 21, choline; 22, valine; 23, 3-hydroxybutyrate (3-HB); 25, isoleucine; 26, leucine; 28, lysine; 32, phosphocholine; 37, tyrosine; 38, histidine; 39, phenylalanine; 40, bile acid; 41, oxidized glutathione (GSSG); 43, uridine; 44, glycogen; 45, adenosine; 46, adenine; 48, adenosine triphosphate (AMP); 49, niacinamide; 50, inosine; 51, glutamate; 52, ATP; 53, uracil; 54, methionine.

We analyzed the metabolic characteristics of the liver obtained from all infected mice. The levels of 23 metabolites significantly changed in liver tissues obtained from mice in the SJ group compared to those obtained from control mice (Fig. 4A and Supplementary Table S3), including an increase in the levels of a range of amino acids, TMAO, choline metabolites, uridine, adenine, inosine and uracil, and decreased levels of succinate, 3-hydroxybutyrate (3-HB), bile acid, GSSG, adenosine, niacinamide, AMP and ATP. Levels of 16 metabolites significantly changed in liver tissues of mice in the CI group compared to controls (Fig. 4B and Supplementary Table S3). The levels of several amino acids, TMAO, succinate and uridine in the coinfected group also reverted back to the levels comparable to controls (Fig. 4B).

### Metabolic changes in serum associated with coinfection

**Figure 5:**
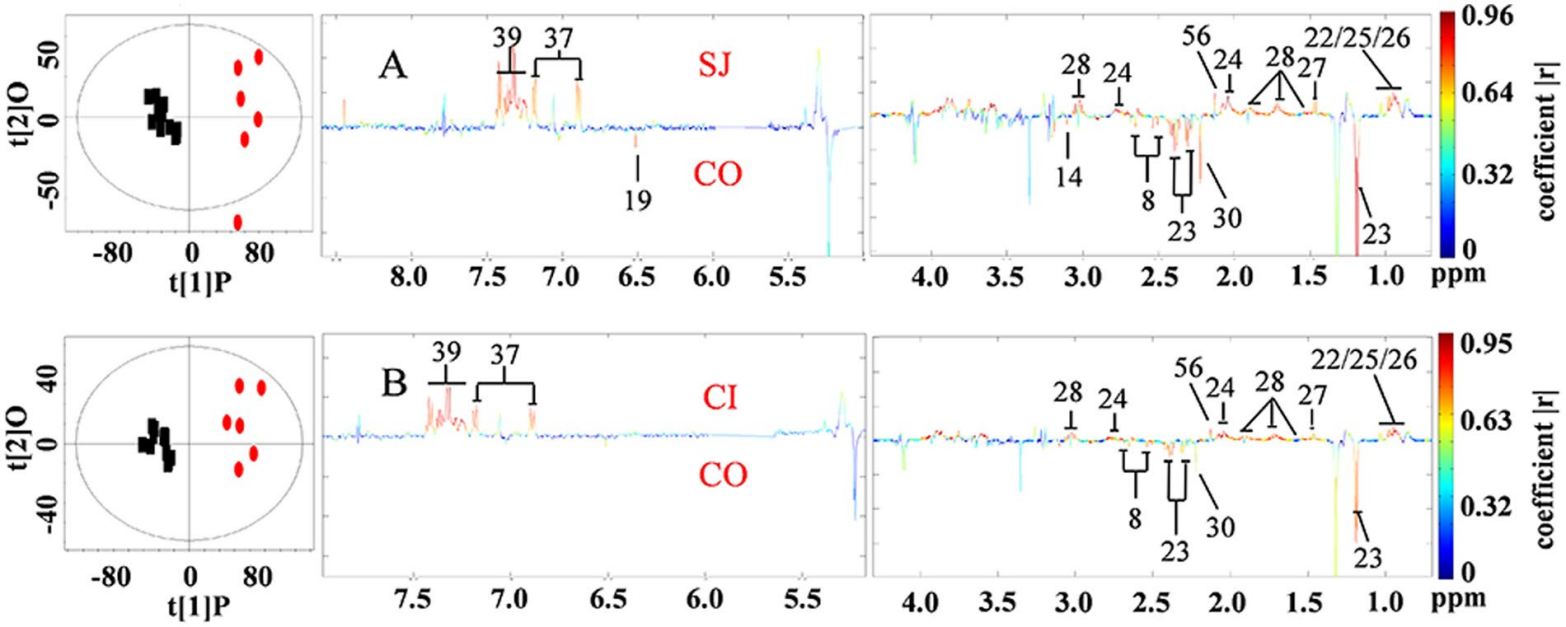
Scores plots and corresponding color-coded coefficient plots. These plots derived from O-PLS-DA of ^1^H NMR spectra obtained from serum of uninfected control mice (CO), *S. japonicum* infected mice (SJ), *S. japonicum and S. typhimurium* coinfected mice (CI). Signals upwards, increase compared with the control; signals downwards, decrease compared with the control. Keys: 8, citrate; 14, malonate; 19, fumarate; 22, valine; 23, 3-hydroxybutyrate; 24, lipids; 25, isoleucine; 26, leucine; 27, alanine; 28, lysine; 30, acetone; 37, tyrosine; 39, phenylalanine; 56, O-acetylglycoproteins.

In serum, we found that elevated levels of a range of amino acids, *O*-acetylglycoproteins, lipids and decreased levels of citrate, malonate, fumarate and 3-HB, were associated with *S. japonicum* single infection (Fig. 5A and Supplementary Table S4). Metabolic alterations associated with coinfection (CI) were similar to those found in the single infection (SJ) and only two metabolites (fumarate, malonate) reverted back to the levels comparable to controls, which is fewer than the results obtained from urine and liver tissues (Fig. 5B and Supplementary Table S4), where many metabolites altered by single infection were returned to the level comparable to those of controls.

### IL-4 and IFN-γ in serum of coinfection

**Figure 6:**
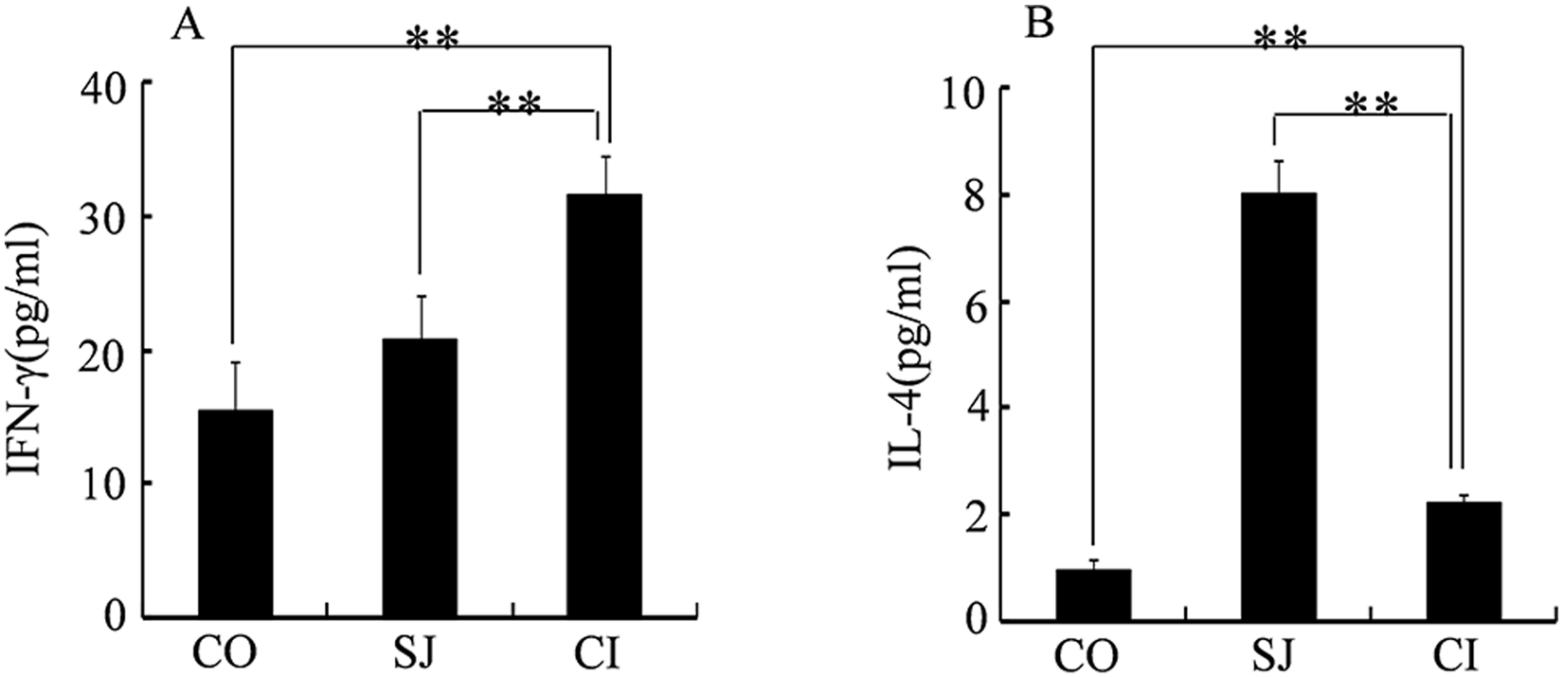
Concentrations (pg/mL) of IFN-γ (A), IL-4 (B) in serum. CO, control mice; SJ, mice infected with *S. japonicum* and CI, mice coinfected with *S. japonicum* and *S. typhimurium*. The statistically significant differences were calculated using independent samples T test with SPSS 13.0 and error bars represent SEM, **p < 0.01.

We further evaluated the impact of coinfection on cytokines in mice. Single infection with *S. japonicum* induced elevations of IFN-γ and coinfection further elevated the levels of IFN-γ (Fig. 6A). However, while a single infection of *S. japonicum* resulted in increased level of IL-4, a decrease in IL-4 was observed in mice of the CI group (Fig. 6B).

## Discussion

In contrast to laboratory animals, humans and other animals in nature are often simultaneously exposed to a large number of acute or chronic infections caused by viruses (HIV, HBV, influenza virus)^36, 37^, bacteria (*Haemophilus influenzae*, *S*. *typhimurium*, *Staphylococcus aureus*)^38^ or helminths (*Plasmodium falciparum*, *S. japonicum*)^39^. Many of these pathogens could be present in the same host concurrently, thereby affecting severity of the infection and disrupting normal metabolism of the host. For this reason, investigation on coinfection is of great importance.

One of the most important findings of the current investigation is that coinfection with *S. typhimurium* (ATCC14028) reduces worm burden and improves the survival rate of *S. japonicum* infected mice (Fig. 1). This is surprising since wild-type *S. typhimurium* (ATCC14028) is generally lethal to rodents^40^, but when infected in mice previously inoculated with *S. japonicum*, it can be beneficial to the host. The improved survival rate is likely due to reduction in the numbers of adult schistosomes and eggs accumulated in the liver and intestinal tissues (Figs 1A,B and 2). The pathogenesis of chronic schistosomiasis is mostly due to the eggs laid by paired adult schistosome that is trapped in the liver^41^. In normal cases, a pair of adult *S. japonicum* produces approximately 3000 eggs per day, which will be transported into the liver and intestine where they inhabit^42^. The soluble proteinase secreted by the eggs initiates host immune responses and provokes the typical eosinophilic inflammatory and subsequent granulomatous reaction, and eventually leads to liver fibrosis^19^. This process in normal circumstances is irreversible even with drug treatment since drugs only kill adult worms but cannot remove eggs that have already deposited in tissues. However, our results showed that co-infecting mice with *S. typhimurium* could potentially reverse the process of schistosomiasis by not only reducing number of adult worms but also reducing the numbers of eggs trapped in liver and intestinal tissues.

**Figure 7:**
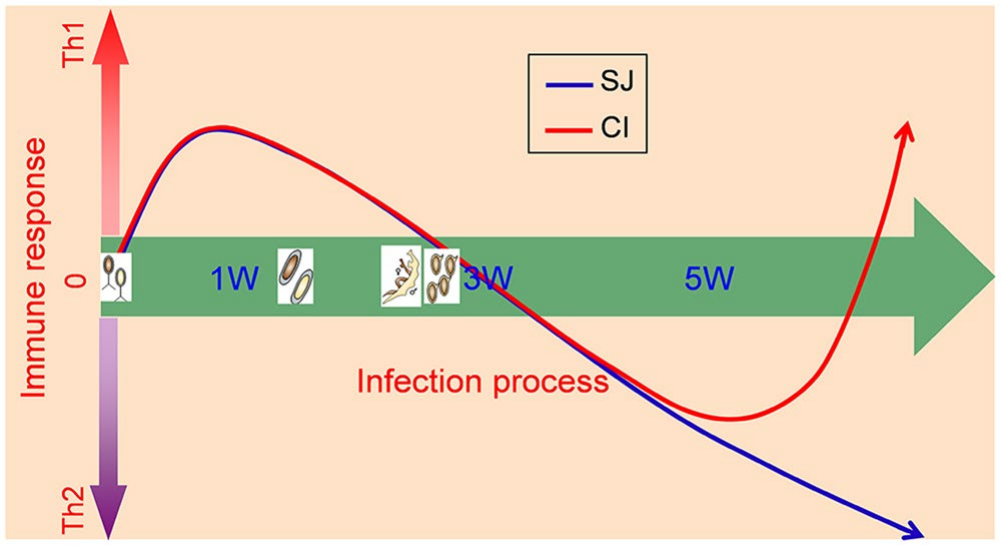
Schematic graph showing the change of immune response of mice to different infections. SJ, immune response of mice infected by *S. japonicum* only; CI, immune response of mice infected by *S. japonicum* firstly then by *S. typhimurium* ATCC14028 five weeks later. Th1, type 1-T helper cell polarized immune response; Th2, type-2 T helper cell polarized immune response. 1 W, 3 W, 5 W, different time points after infection.

The complex immune interaction between the two pathogens within the host could play an important role in the observed phenomenon. The early lifecycle of *S. japonicum* induces the host to develop Th1-polarized cellular immunity in response to *S. japonicum* antigens^43^. After 3 weeks, when paired adult *S. japonicum* worms begin to lay eggs, egg antigens trigger the host immune response towards Th2-mediated humoral immunity^44^. In contrast, the immune response to *S. typhimurium* infection is predominantly Th1-polarized^45, 46^. In our investigation, *S. typhimurium* infection was introduced after five weeks of *S. japonicum* infection, during which time the host’s immune response was Th2-polarized. However, the level of Th2-type cytokine IL-4 in serum decreased sharply while the Th1-type cytokine IFN-γ increased (Fig. 6) nine days after coinfection with *S. typhimurium*. This indicates that *S. typhimurium* infection had stimulated the host immune response to re-establish Th1 polarization. This immune transition coincided with clearance of adult worms and schistosome eggs, indicating that re-establishment of Th1 polarized immune response in host mice could ameliorate schistosomiasis, and most importantly, is associated with reduced organ damage caused by schistosome eggs (Fig. 7). Th1 polarized immune response induced by malaria infection was used for treating neurosyphilis at early twenty century by Julius Wagner-Jauregg who was awarded to the Nobel Prize^47, 48^. Previous studies have found that attenuated *S. typhimurium* carrying Schistosome-antigen can provide high levels of protection against Schistosome infection in a mouse model^49^. The mechanism of the protection effect could be partly similar to our current investigation. The coinfection associated amelioration effects to organ and system, which could be indicated by the restoration of these metabolite levels in the coinfected mice.

The liver is one target organ of *S. japonicum*. In our investigation, we observed significant changes in levels of metabonomes within the liver of mice with *S. japonicum* infection, in particular, the accumulated amino acids, which has been frequently documented^50–53^. Accumulation of amino acids in the liver indicates an *S. japonicum*-associated liver injury. We showed that coinfection with *S. typhimurium* could ameliorate liver injury as suggested by several of amino acids being lapsed to the levels equivalent to controls (Fig. 4 and Supplementary Table S3). Regained function of amino acid metabolism of liver after coinfection could also been manifested in the increased levels of 2-keto-3-methy-valerate in urine of coinfected mice (Supplementary Table S2), a catabolic product of isoleucine. Another important metabolic alteration associated with *S. japonicum* is suppression of the TCA cycle^52, 53^. In our study, we observed decreased levels of succinate in the liver of *S. japonicum* infected mice as well as decreased levels of fumarate in serum of *S. japonicum* infected mice, which are consistent with previous investigations^52, 53^. The depressed TCA cycle has been reverted by introducing secondary infection with *S. typhimurium*. This notion is supported by the leveling of these TCA cycle intermediates observed in the coinfected mice (Supplementary Table S2 and Table S4). Suppressed pyrimidine metabolism has been previously associated with schistosomiasis^52^, which is consistent with our findings, since there were accumulated levels of uridine and 3-ureidopropionate measured in the liver and urine of *S. japonicum* infected mice, respectively. However, it was unclear whether coinfection of *S. japonicum* with *S. typhimurium* restored pyrimidine metabolism to levels comparable to control mice, since we only noted restored levels of uridine, but not 3-ureidopropionate in liver (Supplementary Table S2 and Table S3). Given that urine samples were collected on Day 5 and Day 7 post coinfection, whereas liver samples were obtained on Day 9 post infection, the time difference between the collections may have contributed towards the discrepancies observed.

In conclusion, our study presents strong evidence that coinfection of mice with established schistosomiasis with a secondary pathogen, *S. typhimurium*, could ameliorate the severity of schistosomiasis. The beneficial effects of coinfection were evident due to the reduced burden of *S. japonicum* worms, associated increased survival rate of mice, and restored metabolic function. These effects are likely associated with a shift from a Th2 to Th1 polarized immune response, likely stimulated by the additional *S. typhimurium* infection. Our findings have thus highlighted the importance and complexity of poly-pathogenic infections. Our results have further suggested that stimulating certain immune responses with a second pathogen maybe used for treating certain debilitating diseases, such as schistosomiasis, which could potentially open an alternative avenue for disease control.

## Methods

### Animal experimental procedure

*S. typhimurium* ATCC14028 was cultured aerobically at 37 °C in Luria-Bertani (LB) broth (Sigma Aldrich) overnight and recovered by centrifugation at 10,000 *g* for 1 minute. The recovered cells were washed twice with sterile saline and re-suspended to the final concentration (10^9^ CFU/mL).

Oncomelanias carrying *S. japonicum* cercariae were purchased from Jiangsu Institute of Parasitic Diseases. *S. japonicum* cercariae were released from oncomelanias, which were immersed in dechlorinated water under fluorescent lamp at 26 °C for 6 hours. The numbers of cercariae were counted in small water drops on cover slips under a dissecting microscope.

Six-week old specific pathogen free (SPF) female BALB/c mice were purchased from Vital River Laboratory (Beijing, China) and three weeks of acclimation were allowed before experiments were performed. Mice were housed in well ventilated plastic cages under controlled conditions with free access to rodent food and water (temperature, 22 °C; humidity, 60%; light-dark cycle, 12 h-12 h in SPF animal experimental facility of Wuhan Institute of Physics and Mathematics, CAS).

A total of 36 mice were randomly divided into three groups with each group of 12 mice: control group (CO), single infected group with *S. japonicum* (SJ) and co-infected group with *S. japonicum* followed with *S. typhimurium* (CI). Mice in single infected group with *S. japonicum* alone and co-infected group were inoculated with eighty *S. japonicum* cercariae each via abdominal skin, and mice in the CI group were further infected with *S. typhimurium* ATCC14028 (8 × 10^4^CFU/mL, 0.3 mL per mouse) via gavage after five weeks of *S. japonicum* infection^52, 54^. Mice were sacrificed at 9 days after the second infection. Mice in control group and single infected cases were terminated at the same time as mice in the co-infected group. A urine sample was collected from each mouse at one day before and after inoculation, and at different time intervals (day 1, 3, 5, 7) after the second infection. Urine sample collections were carried out between 8:30–12:00 to avoid potential metabolic variation owning to diurnal rhythm. Adult schistosomes were collected after sacrifice of animals by perfusing the vasculature of mice with ice cold saline solution containing sodium heparin (25 IU/mL)^55^. In addition, worms resident in mesenteric veins were isolated. The collected worms were placed in ice cold saline solution and counted. Schistosome eggs trapped in liver tissues were also counted after digesting liver tissues with 4% KOH at 37 °C overnight^49^. Serum and liver tissue samples were collected at the same time. All samples were immersed in liquid nitrogen immediately after collection and stored at −80 °C until further analysis.

### Ethics statement

All animal experimental procedures were performed in strict accordance with the National Guidelines for Experimental Animal Welfare (People’s Republic of China, 2006) and were approved by the Animal Welfare Committee of Wuhan Institute of Physics and Mathematics, Chinese Academy of Sciences (Permission No. S-051-10-04-OU).

### Measurement of IL-4 and IFN-γ in serum and histopathology

The concentrations of IL-4 and IFN-γ in serum were quantified by IL-4 high sensitive ELISA kits (BMS613HS, eBioscience) and IFN-γ immunoassay kits (MIF00, R&D Systems Inc.) respectively. All measurements were performed according to the respective manufacturer’s protocol.

For histological examination, liver and intestine samples were fixed in 10% buffered neutral formalin, embedded in paraffin, and serially sectioned. Tissue sections were stained with hematoxylin and eosin. Images were captured using a Nikon E100 upright microscope.

### ^1^H NMR spectroscopy

100 µL urine sample was mixed with 400 µL water (10% D_2_O) and 50 µL phosphate buffer (1.5 M, pH = 7.4) containing 0.1% sodium 3-(trimethylsilyl) propionate-2,2,3,3-*d*_4_ (TSP) as chemical shift reference and 0.1% NaN_3_ as aseptic agent^56^. After vortex and centrifugation at 16,000 *g*, 4 °C for 10 minutes, 500 µL supernatant was transferred into a 5 mm NMR tube.

Serum sample (200 µL) was mixed with 400 µL of phosphate buffer saline solution (45 mM, pH = 7.4, 50% D_2_O). The mixture was centrifuged at 11,000 *g*, 4 °C for 10 minutes and 550 µL supernatant was transferred into a 5 mm NMR tube.

Liver samples were extracted by mixing 50 mg tissue with 0.6 mL cold mixture of methanol and water (2:1, v:v), and homogenized by a tissuelyser for 90 seconds at 20 Hz followed by ultrasonication for three one-minute sessions with one minute interval. Supernatants were collected after centrifugation at 11,000 *g*, 4 °C for 10 minutes. The extraction procedure was repeated three times and supernatants from the same samples were combined. Methanol was removed by speed vacuum and extractions were freeze-dried. The resulting powder was reconstituted in 0.6 mL phosphate buffer (0.1 M, pH = 7.4, 50% D_2_O, 0.05% TSP and 0.1% NaN_3_) and 550 µL supernatant was transferred into a 5 mm NMR tube following centrifugation.

^1^H NMR spectra of urine and liver extracts were acquired at 298 K using Bruker AVIII 600 MHz NMR spectrometer (Bruker Biospin, Germany) with cryogenic probe, operating at proton frequency of 600.13 MHz. The first increment of NOESY pulse sequence (recycle delay-90°-*t*_*1*_-90°-*t*_*m*_-90°-acquisition) with water suppression was employed. ^1^H NMR spectra of serum were acquired at 298 K on Bruker AVII 500 MHz NMR spectrometer with broad band inverse detection probe, operating at 500.13 MHz proton frequency. Spin-spin relaxation edited ^1^H NMR experiment using Carr-Purcell-Meiboom-Gill (CPMG) pulse sequence with water saturation was performed for serum. Two-dimensional NMR spectra (^1^H-^1^H COSY and TOCSY, ^1^H-^13^C HSQC and HMBC) were acquired for selected samples to assist the spectral assignment.

### NMR spectral data processing and multivariate pattern recognition analysis

^1^H NMR spectra were manually corrected for phase and baseline deformation using Topspin software package (V2.0, Bruker Biospin, Germany). Spectra of urine and liver extracts were referenced to TSP (δ, 0 ppm), whereas serum spectra were calibrated to the low field peak (δ, 5.233 ppm) of the doublets belonging to α-glucose. The spectra ranging from δ 0 to δ 10 ppm were integrated with an equal width of 0.002 ppm for urine and liver extracts and 0.004 ppm for serum using the AMIX package (V3.8, Bruker Biospin, Germany). Water regions were removed and normalization to total intensity of the spectrum was carried out for urinary and serum spectra, while normalization to the wet weight was performed for the spectra of liver extracts. Principal component analysis (PCA) and orthogonal-projection to latent structures discriminant analysis (O-PLS-DA) of spectral data were conducted using SIMCA-P^+^ software package (V12, Umetrics, Sweden)^57, 58^. Unit variance (UV) scaling for multivariate statistics was employed. The model quality was indicated by corresponding parameters, such as Q^2^and R^2^, denoting predictability and interpretability of the model, respectively. O-PLS-DA models were validated using a 7-fold cross-validation method, and further ensured with CV-ANOVA test^59^. The discrimination significance *p* values of given metabolites were set <0.05. For visualization, the loadings were back-transformed and plotted with color-coded coefficients for each variable using an in-house developed MATLAB script^60^.

## Electronic supplementary material

Supplementary information
