# Supplementary material for: Salmonella typhimurium Infection Reduces Schistosoma japonicum Worm Burden in Mice
Source: Sci Rep. 2017 May 2;7:1349. doi: 10.1038/s41598-017-00992-1 (PMC5430953; doi:10.1038/s41598-017-00992-1)
Supplement: Supplementary file 1 — Supplementary information [file 41598_2017_992_MOESM1_ESM.pdf]

Salmonella typhimurium Infection Reduces *Schistosoma japonicum* Worm Burden in Mice

Xiaoyang Zhu[1], Lu Chen[1], Junfang Wu[1], Huiru Tang[2,*], Yulan Wang[1,3,*]

[1]CAS Key Laboratory of Magnetic Resonance in Biological Systems, State Key Laboratory of Magnetic Resonance and Atomic and Molecular Physics, National Centre for Magnetic Resonance in Wuhan, Wuhan Institute of Physics and Mathematics, the Chinese Academy of Sciences, Wuhan 430071, China

[2]State Key Laboratory of Genetic Engineering, Zhongshan Hospital and School of Life Sciences, Fudan University, Collaborative Innovation Centre for Genetics and Development, Shanghai International Center for Molecular Phenomics, Shanghai 200433, China

[3]Collaborative Innovation Center for Diagnosis and Treatment of Infectious Diseases, Zhejiang University, Hangzhou 310058, China

*, corresponding author

To whom correspondence should be addressed:

Yulan Wang, Email, yulan.wang@wipm.ac.cn; tel, +86-27-87197143; fax:+86-27-87199291

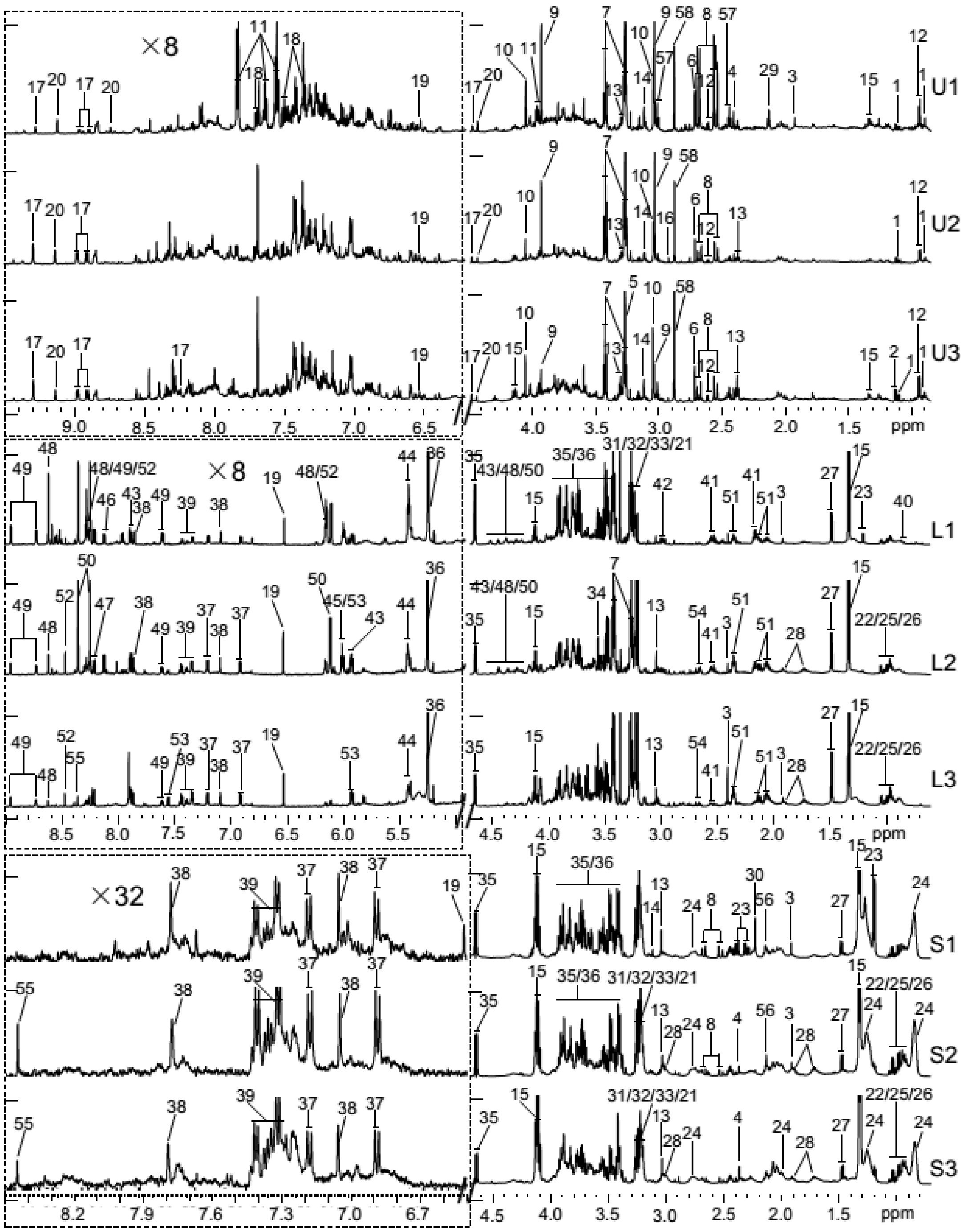

Supplementary Figure S1. Typical ^{1}H NMR spectra of urine (U), liver (L) and serum (S). Samples were obtained from an uninfected control mouse (1), a mouse (2) infected by *S. japonicum* and a mouse (3) coinfected by *S. japonicum* with *S. typhimurium*. The low magnetic field spectral regions were vertically magnified 8, 8, 32 times compared with the high magnetic field region. Keys can be found in Supplementary Table S1.

Supplementary Table S1. Table of assignment of the metabolites in urine, serum, liver.

keys	metabolites	moieties	δ [1]H (ppm) and multiplicity
1	2-keto-3-methy-valerate	CH_3, β-CH_3, β-CH, γ-CH, γ-CH	0.90(t), 1.10 (d), 2.93(m), 1.46(m), 1.70(m)
2	2-keto-isovalerate	γ-CH_3, β-CH	1.13(d), 3.03(m)
3	acetate	CH_3	1.93(s)
4	succinate	CH_2	2.41 (s)
5	TMAO	CH_3	3.27 (s)
6	DMA	CH_3	2.72(s)
7	taurine	CH_2-SO_3, CH_2-NH	3.27(t), 3.43(t)
8	citrate	CH_2(1/2), CH_2(1/2)	2.56(d), 2.69(d)
9	creatine	CH_3, CH_2	3.04(s), 3.94(s)
10	creatinine	CH_3, CH_2	3.05(s), 4.06(s)
11	hippurate	CH_2, H3, H5, H4, H2, H6	3.97 (d), 7.55(t), 7.64(t), 7.84(d)
12	2-keto-isocaproate	δ-CH_3, β-CH_2	0.94(d), 2.62(d)
13	3-UP	α-CH_2, β-CH_2	2.38(t), 3.31(q)
14	malonate	CH_2	3.12(s)
15	lactate	β-CH_3, α-CH	1.34(d), 4.12(q)
16	DMG	CH_3, CH_2	2.93(s), 3.72(s)
17	NMN	CH_3, H5, H4, H6, H2	4.48(s), 8.18(d), 8.90(d), 8.97(d), 9.29(s)
18	indoxyl sulfate	H5, H6, H2, H7, H4	7.21(t), 7.28(t), 7.36 (s), 7.51(d), 7.71(d)
19	fumarate	CH	6.53(s)
20	trigonelline	CH_3, H4, H3,5, H1	4.44(s), 8.076(m), 8.81(m), 9.13(s)
21	choline	$N(CH_3)_3$, N-CH_2, O-CH_2	3.20 (s), 3.50 (t), 4.09(m)
22	valine	CH_3, CH_3, CH- NH_2, CH	0.99(d), 1.04(d), 2.27(m), 3.62(d)
23	3-HB	CH_3, CH, β-CH_2(1/2), β-CH_2(1/2)	1.19(d), 4.15(m), 2.30(m), 2.39(m)
24	lipids	CH_3, $(CH_2)_n$, CH_2-C=C CH_2-C=O, C-CH_2-C=, -CH=CH-	0.85(m), 1.27(m), 2.00(m), 2.23(m), 2.74(m), 5.30(m)
25	isoleucine	δ-CH_3, β-CH_3, γ-CH_2(1/2) γ-CH_2(1/2), β-CH, α-CH	0.93(t), 1.00(d), 1.25(m), 1.46(m), 2.00(m), 3.66(d)
26	leucine	δ-CH_3, δ-CH_3, γ-CH, β-CH_2, α-CH	0.95(d), 0.96(d), 1.70(m), 1.74(m), 3.76(t)
27	alanine	β-CH_3, α-CH	1.47(d), 3.78(q)
28	lysine	γ-CH_2, δ-CH_2, β-CH_2, ε-CH_2, α-CH_2	1.49(m), 1.78(m), 1.92(m), 3.00(t), 3.76(t)

29	*N*-acetylglutamic acid	CH_3, CH_2,	2.13(s), 2.79(t),
		CH_2, CH	1.84(m), 4.03(t)
30	acetone	CH_3	2.22(s)
31	betaine	$N(CH_3)_3$, N-CH_2	3.25(s), 3.91(s)
32	PC	$N(CH_3)_3$, N-CH_2, O-CH_2	3.22(s), 3.62(t), 4.24(t)
33	GPC	$N(CH_3)_3$, N-CH_2,	3.23(s), 3.62(m),
		O-CH_2, P-CH_2, P-CH_2	3.67 (t), 3.91(m), 4.32(t)
34	glycine	CH_2	3.55(s)
35	β-glucose	H2, H4, H3,	3.24(m), 3.40(m), 3.46(m),
		H5, CH_2-C6, H1	3.76(dd), 3.90(dd), 4.65(d)
36	α-glucose	H4, H2, H3,	3.40(m), 3.54(dd), 3.71(m),
		H5, H6, H1	3.82(m), 3.83(m), 5.23(d)
37	tyrosine	β-CH_2(1/2), β-CH_2(1/2),	3.05(m), 3.14(m),
		α-CH	3.96(m),
		H3, H5; H2, H6	6.89(d), 7.19(d)
38	histidine	β-CH_2(1/2),	3.07(d),
		β-CH_2(1/2), α-CH	3.13(d), 3.96(dd),
		H4, H2	7.05(s), 7.78(s)
39	phenylalanine	β-CH_2(1/2),	3.11(m),
		β-CH_2(1/2), α-CH,	3.28(m), 3.98(m),
		H2, H6, H4, H3, H5	7.18(d), 7.32(m), 7.41(m)
40	bile acid	CH_3, CH_3	0.74(s), 0.84(s)
41	GSSG	Glu: β-CH_2, γ-CH_2, α-CH_2	2.17(m), 2.54(q), 3.78(m),
		Cys: β-CH_2, β-CH_2, α-CH_2	2.98(m), 3.32(m), 4.76(m)
42	asparagine	β-CH_2(1/2),	2.88(dd),
		β-CH_2(1/2), α-CH	2.96(dd), 4.00(m)
43	uridine	CH_2(1/2), CH_2(1/2),	3.80(m), 3.90(m),
		H(d), H(c)	4.12(m), 4.24(t),
		H(b), H(a),	4.36(t), 5.91(d),
		H5, H6	5.93(d), 7.89(d)
44	glycogen	C-OH, C-OH,	3.42(m), 3.59(m),
		CH_2, C-OH, O-CH-O	3.79(m), 3.98(br,s), 5.43(br,s)
45	adenosine	CH_2, O-CH,	3.86(m), 4.29(q),
		HO-CH, HO-CH	4.42(dd), 4.73(s),
		O-CH-N, H3, H7	5.99(d), 8.12(s), i8.28(s)
46	adenine	H2, H7	8.10(s), 8.11(s)
47	hypoxanthine	H2, H7	8.20(s), 8.22(s)
48	AMP	CH_2, O-CH,	4.03(dd), 4.38(dd),
		HO-CH, HO-CH	4.52(dd), 4.78(d),
		O-CH-N, H3, H7	6.15(d), 8.27(s), 8.60(s)
49	niacinamide	H3, H4,	7.60(dd), 8.695(dd),
		H2, H1	8.705(dd), 8.95(s)
50	inosine	CH_2(1/2), CH_2(1/2),	3.91(dd), 3.93(dd),
		H5', H4'	4.29(dd), 4.45(dd),

		H3', H2', H8, H2	4.79(s), 6.11(d), 8.25(s), 8.36(s)
51	glutamate	β-CH_2, γ-CH_2, α-CH	2.10(m), 2.34(m), 3.76(m)
52	ATP	1-CH', 2-CH, 7-CH	6.15(d), 8.54(s), 8.27(s)
53	uracil	CH, CH	5.81(d), 7.55(d)
54	methionine	α-CH, β-CH_2, γ-CH_2, δ-CH_3	3.87(t), 2.15(m), 2.65(t), 2.14(s)
55	formate	CH	8.46(s)
56	*O*-acetylglycoproteins	CH_3	2.14(s)
57	2-keto-glutarate	β-CH_2, γ-CH_2	2.45(t), 3.01(t)
58	trimethylamine	CH_3	2.88(s)

s, singlet; d, doublet; t, triplet; q, quarter; m, multiplet; dd, doublet of doublet; br.s, broad singlet. TMAO, trimethylamine-*N*-oxide; DMA, dimethylamine; 3-UP, 3-ureidopropionate; DMG, dimethylglycine, NMN, *N*-methylnicotinamide; 3-HB, 3-hydroxybutyrate; PC, phosphocholine; GPC, glycerophosphocholine; GSSG, oxidized glutathione; AMP, adenosine monophosphate; ATP, adenosine triphosphate.

Supplementary Table S2 Urinary metabolites contribute to the discrimination between different groups of mice at two post infection time points.

metabolites (key)	δ (ppm)	SJ vs CO[a]	CI vs CO[a]	SJ vs CO[b]	CI vs CO[b]
2-keto-3-methy-valerate(1)	0.90, 1.10	-	+0.84	-	-
2-keto-isovalerate (2)	1.13, 3.03	+0.78	+0.87	-	-
succinate (4)	2.41	-	-	-0.79	-0.71
TMAO (5)	3.27	-0.73	-0.73	-0.75	-0.78
taurine (7)	3.27, 3.43	+0.90	-	+0.82	-
citrate (8)	2.56, 2.69	-	-	-0.71	-
creatine (9)	3.04, 3.94	+0.83	-	+0.81	-
creatinine (10)	3.05, 4.06	+0.88	+0.75	-0.84	-
hippurate (11)	3.97, 7.55, 7.64, 7.84	-0.96	-0.87	-0.88	-0.60
2-keto-isocaproate (12)	2.61	-0.80	-0.75	-0.78	-0.80
3-UP (13)	2.38, 3.31	+0.77	+0.73	+0.93	+0.79
DMG (16)	2.93, 3.72	+0.96	-	+0.85	-
N-methylnicotinamide (17)	4.48, 8.90, 8.97, 9.29	+0.90	-	+0.89	+0.76
indoxyl sulfate (18)	7.21, 7.28, 7.51, 7.36	-	-	-0.85	-0.69
fumarate (19)	6.53	-0.72	-0.73	-0.67	-
trigonelline (20)	4.44, 8.08, 8.81, 9.13	-0.78	-0.81	-0.81	-0.61
N-acetylglutamic acid (29)	2.13, 2.79, 4.03	-0.70	-0.78	-	-

TMAO, trimethylamine-*N*-oxide; 3-UP, 3-ureidopropionate; DMG, dimethylglycine.

CO, group of mice serving as control without being infected; SJ, mice infected with 80 cercariae of *S. japonica* only; CI, mice infected with 80 cercariae of *S. japonica* followed by *S. typhimurium* infection at 5 weeks later; a, time point, five days after coinfection; b, time point, seven days after coinection.

Supplementary Table S3 Metabolites of liver with significant contributions to discrimination between different groups of mice.

metabolites (key)	δ (ppm)	SJ v CO	CI v CO
succinate (4)	2.41	-0.70	-
TMAO (5)	3.27	+0.83	-
taurine (7)	3.27, 3.43	-	-0.67
choline (21)	3.20, 3.50, 4.09	+0.89	+0.90
valine (22)	0.99, 1.04,	+0.78	-
3-HB (23)	1.19	-0.75	-0.72
isoleucine (25)	0.93, 1.00	+0.82	-
leucine (26)	0.95, 0.96, 1.70, 1.74	+0.89	+0.7
lysine (28)	1.49, 1.78, 1.92	+0.87	-
phosphocholine (32)	3.22, 3.62, 4.24	+0.73	+0.93
tyrosine (37)	6.89, 7.19	+0.87	-
histidine (38)	7.05, 7.78	-	-0.84
phenylalanine (39)	7.18, 7.32, 7.41	+0.90	-
bile acid (40)	0.74, 0.84	-0.95	-0.96
GSSG (41)	2.17, 2.54, 3.79, 2.97, 3.32	-0.83	-0.91
uridine (43)	5.91, 5.93, 7.89	+0.89	-
glycogen (44)	3.42, 3.59, 3.79, 3.98, 5.43	-	+0.82
adenosine (45)	5.99	-0.93	-0.91
adenine (46)	8.10, 8.11	+0.92	-0.77
AMP (48)	4.03, 4.38, 4.52, 6.15, 8.27, 8.60	-0.94	-0.96
niacinamide (49)	7.60, 8.69, 8.71, 8.95	-0.93	-0.96
inosine (50)	4.29, 4.45, 4.79, 6.11, 8.25, 8.36	+0.87	-0.71
glutamate (51)	2.10, 2.34, 3.76	+0.86	-
ATP (52)	6.15, 8.54, 8.27	-0.96	-0.96
uracil (53)	5.81, 7.55	+0.94	+0.99
methionine (54)	3.87, 2.15, 2.65, 2.14	+0.85	-

TMAO, trimethylamine-*N*-oxide; 3-HB, 3-hydroxybutyrate; AMP, adenosine monophosphate; ATP, adenosine triphosphate； GSSG, oxidized glutathione.

CO, group of mice serving as control without being infected; SJ, mice infected with 80 cercariae of *S. japonica* only; CI, mice infected with 80 cercariae of *S. japonica* followed by *S. typhimurium* ATCC14028 infection 5 weeks later.

Supplementary Table S4 Metabolites of serum with significant contributions to discrimination between different groups of mice.

metabolites (key)	δ (ppm)	SJ v CO	CI v CO
citrate (8)	2.56, 2.69	-0.85	-0.78
malonate (14)	3.12	-0.81	-
fumarate (19)	6.53	-0.84	-
valine (22)	0.99, 1.04	+0.77	+0.80
3-hydroxybutyrate (23)	1.19, 2.30, 2.39	-0.90	-0.76
lipids (24)	1.27, 2.00, 2.23, 2.74	+0.95	+0.86
isoleucine (25)	0.93, 1.00, 1.25	+0.89	+0.88
leucine (26)	0.95, 0.96	+0.78	+0.79
alanine (27)	1.47, 3.78	+0.81	+0.76
lysine (28)	1.49, 1.78, 1.92	+0.76	+0.77
O-acetylglycoproteins (56)	2.14	+0.84	+0.84
acetone (30)	2.22	-0.77	-0.76
tyrosine (37)	6.89, 7.19	+0.78	+0.81
phenylalanine (39)	7.18, 7.32, 7.41	+0.82	+0.82

CO, group of mice serving as control without being infected; SJ, mice infected with 80 cercariae of *S. japonica* only; CI, mice infected with 80 cercariae of *S. japonica* followed by *S. typhimurium* ATCC14028 infection 5 weeks later.

Supplementary Table S5 Model summary of O-PLS-DA of ^{1}H NMR spectra of urine, liver and serum at different time point obtained from different group of mice.

urine time points	SJ vs CO			CI vs CO		
	R^2X	Q^2	*p*(cv)	R^2X	Q^2	*p*(cv)
ada	0.36	0.22	>0.05	0.34	0.37	>0.05
5w	0.40	0.95	<0.01	0.41	0.85	<0.01
5w+1d	0.44	0.92	<0.01	0.42	0.66	<0.01
5w+3d	0.43	0.97	<0.01	0.37	0.84	<0.01
5w+5d	0.50	0.93	<0.01	0.42	0.77	<0.01
5w+7d	0.47	0.88	<0.01	0.42	0.75	<0.01

	liver		serum	
	SJ vs CO	CI vs CO	SJ vs CO	CI vs CO
R^2X	0.43	0.60	0.40	0.35
Q^2	0.95	0.89	0.73	0.56
p(cv)	<0.01	<0.01	<0.01	<0.05

CO, group of mice serving as control without being infected; SJ, mice infected with 80 cercariae of *S. japonica* only; CI, mice infected with 80 cercariae of *S. japonica* followed by *S. typhimurium* ATCC14028 infection 5 weeks later. ada, adaptive phase. 5w, 5 weeks after mice of SJ and CI groups infected with 80 cercariae of *S. japonicum*. 5w+1d…5w+7d, time points represent sampling time one day … seven days after *S. typhimurium* infection.
